# Hypoxic Tumor Microenvironment Targeting: Opportunities and Challenges for Pancreatic Cancer Immunotherapy

**DOI:** 10.3390/ijms27093873

**Published:** 2026-04-27

**Authors:** Raefa Abou Khouzam, Shaima Salman, Jerome Thiery, Rania Faouzi Zaarour, Visar Vela, Perparim Limani, Bassam Janji, Salem Chouaib

**Affiliations:** 1 Thumbay Research Institute for Precision Medicine, Gulf Medical University, Ajman 4184, United Arab Emirates; 2 Armstrong Oxygen Biology Research Center and Institute for Cell Engineering, Johns Hopkins University School of Medicine, Baltimore, MD 21205, USA; 3 INSERM UMR 1356, Next-Generation Immuno-Oncology Research and Therapy in Pediatric and Adult Cancer, Gustave Roussy, Faculty of Medicine, University Paris-Saclay, 94805 Villejuif, France; 4 Gastrointestinal Research Unit, Department of General Surgery and Surgical Oncology, Cantonal Hospital Aarau, Tellstrasse 25, CH-5001 Aarau, Switzerland; 5 Swiss Hepato-Pancreato-Biliary (HPB) and Transplantation Center, University Hospital Zurich, Raemistrasse 100, CH-8091 Zurich, Switzerland; 6 Department of Surgery & Transplantation, University Hospital Zurich, Raemistrasse 100, CH-8091 Zurich, Switzerland; 7 Department of Cancer Research, Luxembourg Institute of Health, Tumor Immunotherapy and Microenvironment (TIME) Group, 6A, Rue Nicolas-Ernest Barblé, L-1210 Luxembourg City, Luxembourg

**Keywords:** hypoxia, immunotherapy, immunosuppression, hypoxia alleviation, hypoxia targeting, hypoxia gene signatures, pancreatic cancer

## Abstract

Pancreatic ductal adenocarcinoma (PDAC) remains among the deadliest cancers, with a 5-year survival rate of 13% and broad resistance to therapy. It is driven by severe tumor hypoxia from desmoplasia, aberrant vasculature, and high interstitial pressure. Hypoxia stabilizes hypoxia-inducible factors (HIFs), reshaping the tumor microenvironment (TME) into a nutrient-poor, acidic milieu that fosters immune exclusion and suppression. While immune checkpoint inhibitors (ICIs) have revolutionized treatment, PDAC responses have been negligible. As hypoxia centrally drives PDAC’s ICI-refractory TME, targeted alleviation could offer synergy with ICIs; however, no such combination is being applied in the clinic. One impediment could be the one-size-fits-all approach when investigating hypoxia-modifying therapy. Indeed, using hypoxia gene signatures, we and others have shown that PDAC tumors are not equally hypoxic, with patients having more hypoxic tumors experiencing worse survival and immunosuppressed TME. This review dissects hypoxia’s mechanistic role in PDAC immune evasion and gives an update on the therapeutic advances that directly or indirectly target hypoxia, such as the inhibition of HIFs, hypoxia-activated prodrugs, and vascular and oxygen delivery approaches, with emphasis on their potential to enhance responses to ICIs. It further evaluates the need for hypoxia biomarkers and proposes gene signatures as detection tools to enable precision hypoxia modulation, potentially converting immune-cold PDAC into an ICI-responsive disease.

## 1. Introduction

Pancreatic ductal adenocarcinoma (PDAC) is among the most lethal malignancies, with a five-year survival rate of 13% and broad resistance to cytotoxic and targeted therapies [1,2]. A defining hallmark of PDAC is tumor hypoxia, with tissue oxygen tensions often below 10 mmHg and frequently below 5 mmHg, compared to 40–60 mmHg in normal pancreatic tissue [1,3,4].

This severe hypoxia, which positions PDAC among the most hypoxic of all solid tumors, arises from a unique confluence of factors. In particular, abundant desmoplastic stroma can comprise up to 90% of PDAC tumor volume and consists of activated cancer-associated fibroblasts (CAFs), predominantly pancreatic stellate cells (PSCs), a dense extracellular matrix (ECM) as well as compressed dysfunctional vessels [5,6,7]. This desmoplasia creates a physical barrier to perfusion and generates elevated interstitial fluid pressure that collapses vessels [5,6,7]. The resulting combination of extreme desmoplasia-driven vascular collapse, a high stromal-to-tumor ratio, and rapid tumor proliferation creates a self-perpetuating cycle of severe hypoxia that distinguishes PDAC from most other malignancies [4,5,6,7].

**Figure 1:**
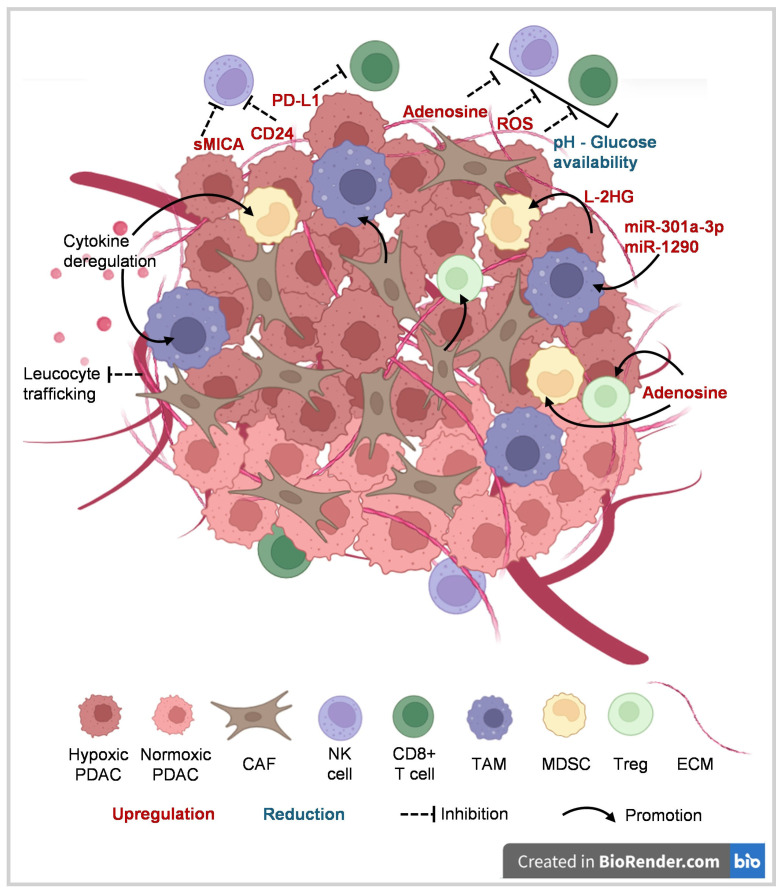
Select mechanisms of hypoxia-mediated immunosuppression in the microenvironment of pancreatic ductal adenocarcinoma. CAF: cancer-associated fibroblasts; ECM: extracellular matrix; L-2HG: L-isoform 2-hydroxyglutarate; MDSC: myeloid-derived suppressor cells; NK: natural killer; PDAC: pancreatic ductal adenocarcinoma; PD-L: programmed cell death protein-ligand 1; ROS: reactive oxygen species; sMICA: soluble major histocompatibility complex class 1-rleated molecule A; TAM: tumor-associated macrophage; Treg: regulatory T cell. (Created in BioRender. AK, R. (2026) https://BioRender.com/n7rmad8).

The hypoxic microenvironment stabilizes hypoxia-inducible factors (HIFs), the master transcriptional regulators of oxygen homeostasis, driving adaptive programs that directly contribute to PDAC’s dismal prognosis [1,8,9,10,11]. HIFs are heterodimeric transcription factors composed of an oxygen-regulated α subunit (HIF-1α or HIF-2α) and a constitutively expressed β subunit (ARNT/HIF-1β) [8,9]. Under normal oxygen levels, HIF-α subunits are hydroxylated by prolyl hydroxylase domain (PHD) enzymes, recognized by the von Hippel–Lindau (VHL) E3 ligase, and targeted for proteasomal degradation. Under hypoxia, PHD activity is inhibited, enabling HIF-α stabilization, nuclear translocation, and transcriptional activation of hundreds of target genes that promote angiogenesis, metabolic rewiring, epithelial-to-mesenchymal transition (EMT), stemness, and metastasis [8,10]. Hypoxia in the tumor microenvironment (TME) further contributes to oxidative stress; regulates the activity of stromal cells, including CAFs; and impacts immune cell infiltration as well as the tumor’s susceptibility to clearance by immune cells (Figure 1). Hypoxia therefore directly shapes the response to therapy, including immunotherapy.

Immune checkpoint inhibitors (ICIs) target negative regulators of cytotoxic T-cell function, including programmed cell death protein-1 (PD-1), its ligand PD-L1, and cytotoxic T-lymphocyte-associated protein-4 (CTLA-4). Anti-PD-1 and anti-PD-L1 antibodies primarily reinvigorate antigen-experienced CD8^+^ T cells, whereas anti-CTLA-4 enhances the priming and activation of naïve T cells. ICIs have produced substantial clinical benefit in melanoma [12], non-small cell lung cancer [13], and triple-negative breast cancer [14]. Meanwhile, their efficacy in PDAC has been disappointing [15], with the only clearly responsive subset comprising fewer than 1% of cases [16]. While some trials have reported improved responses when ICIs are combined with chemotherapy in advanced PDAC [17,18], overall response rates appear comparable to chemotherapy alone [15]. Indeed, PDAC is classically described as an immune-cold tumor, with a TME that is sparsely infiltrated by effector immune cells [19]. Instead, it is enriched in immunosuppressive populations such as M2-polarized macrophages, regulatory T cells (Tregs), and myeloid-derived suppressor cells (MDSCs), which collectively promote tumor survival, proliferation, and dissemination. In contrast, antitumor effectors including CD8^+^ cytotoxic T lymphocytes and natural killer (NK) cells are frequently excluded from the tumor bed or rendered functionally exhausted, with their cytolytic activity severely impaired. A major upstream driver of this immune-excluded, immunosuppressive milieu of PDAC that underlies its poor responsiveness to immunotherapy, is hypoxia.

Because hypoxia is central to PDAC’s resistance to ICIs, its targeting or alleviation may be critical for improving patient outcomes. Therapeutically, hypoxia constitutes an actionable vulnerability, amenable to multifaceted interventions that inhibit HIF signaling, enhance tumor oxygenation, normalize aberrant vasculature, and reprogram the immunosuppressive TME. Preclinical and early clinical data indicate that hypoxia alleviation can synergize with ICIs, but broader clinical translation has been constrained, at least in part, by a lack of robust biomarkers.

This review dissects how hypoxia drives PDAC immunosuppression through HIF-mediated signaling, oxidative stress adaptation, CAF reprogramming, and tumor plasticity. It further evaluates hypoxia-targeting therapeutic strategies, including HIF inhibitors, oxygen delivery agents, and vascular normalization, alongside emerging hypoxia biomarkers, namely gene signatures, to enable precision, biomarker-guided interventions that may convert immune-cold PDAC into an immunotherapy-responsive disease.

## 2. Hypoxia Underpins Immunosuppression and Immune Resistance Limiting Response to Immunotherapy

Hypoxia in PDAC drives defects in both innate and adaptive antitumor immunity [1,19,20]. Extensive evidence demonstrates that hypoxia orchestrates multiple downstream pathways, including metabolic remodeling, cytokine and chemokine signaling, microRNA expression, antigen-presentation and immune checkpoint regulation, thereby establishing an immunosuppressive TME that ultimately limits the efficacy of cancer immunotherapies (Figure 1) [21,22].

Adaptive responses to oxygen deprivation create a nutrient-poor, highly acidic TME that actively enforces immune exclusion and suppression. The stabilization of HIF-1α in hypoxic cells rewires metabolism by upregulating glucose transporters and glycolytic genes [23]. Tumor cells thereby outcompete immune cells for glucose, inhibiting the cytolytic activity of CD8^+^ T cells and NK cells, blocking the polarization of macrophages toward the antitumor M1 phenotype, and impairing dendritic cell maturation and function [24]. HIF-1α also induces transporters that export lactate and hydrogen ions, lowering extracellular pH. This acidic environment further compromises effector leukocyte function yet is tolerated by Tregs [25,26]. Lactate additionally promotes MDSC recruitment and drives macrophage polarization toward an immunosuppressive M2 phenotype [27,28]. The L-isoform of the oncometabolite 2-hydroxygluterate (L-2HG) which is derived from non-canonical LDHA (lactate dehydrogenase A) activity, has been shown to accumulate in a HIF1-dependent manner as a physiological response to hypoxia [29,30] and to be produced by tumor and stromal cells in the pancreas [31]. Importantly, the presence of L-2HG in the microenvironment inhibited the infiltration of CD8+ T-cells by incapacitating their proliferation and migration [31].

Aside from metabolic byproducts, hypoxia impacts immune exclusion by shaping the cytokine and chemokine profile in PDAC. HIF-1α expression positively correlates with CCL2 (C-C Motif Chemokine Ligand 2) levels and increased macrophage infiltration, directly linking hypoxia to protumor myeloid recruitment [32]. In hypoxic PDAC cells, cytokine deregulation, namely the increased expression of IL-6 (interleukin 6) coupled by the reduction in TNF-α (tumor necrosis factor-α) and IFN-γ (interferon-γ) levels, was associated with their reduced susceptibility to CD8+ T-cells [33]. Innate lymphoid cells group 2 (ILC2s) are a highly dynamic cell type whose phenotype and function are controlled by the TME [34]. In that respect hypoxia induced the transition of ILC2 to interleukin 10 (IL10) + ILCregs promoting the formation of an immunosuppressive TME and disease progression in a PDAC patient subgroup [34].

Immunosuppression in PDAC is further accrued at multiple levels by hypoxia-modulated miRNAs (microRNAs), known as hypoxamiRs [20]. The Axis Inhibition Protein 2 (AXIN2)-suppressor, miR-1275 is upregulated in the hypoxic TME of pancreatic cancer [35]. The resulting overexpression of AXIN2 impaired NK cell activity through decreased expression levels of perforin, TNF-α, and IFN-γ, thus advocating the immune escape of PDAC cells [35]. Exosomal miR-301a-3p [36] and miR-1290 [37] were found to stimulate M2 polarization of macrophages in the hypoxic microenvironment of PDAC cells. Similarly, a key hypoxamiR, miR-210, was found to promote M2 polarization when released from pancreatic cancer stem cells in exosomes [38]. Circular RNAs have also been implicated in reconfiguring the immune response. In particular, circ_0000977 was found to downregulate miR-153 in PDAC cells. This in turn upregulated HIF-1α and the protease ADAM10 (a disintegrin and metalloproteinase domain-containing protein 10). ADAM10 resulted in the shedding of mMICA (membrane major histocompatibility complex class 1-rleated molecule A) thus increasing soluble MICA (sMICA), which contributed to immune escape by impairing the function of NKG2D (natural killer group 2, member D) receptor complexes on NK cells [39].

Hypoxia also remodels the vasculature and antigen-presentation machinery in ways that facilitate immune escape. Pathological angiogenesis under hypoxia yields leaky, disorganized vessels that restrict effective leukocyte trafficking, while the altered endothelium downregulates the immune homing receptors required for leukocyte extravasation into the tumor [22]. At the same time, hypoxia suppresses major histocompatibility complex class I (MHC-I) expression through HIF-1α-induced autophagy [40], undermining adaptive immune surveillance. Since MHC-I presents endogenous peptides, including neoantigens, to CD8^+^ T cells, its loss leads to reduced CD8^+^ T cell cytotoxicity, thus diminishing the recognition and clearance of malignant cells.

Beyond antigen presentation, hypoxia promotes immune escape through the upregulation of co-inhibitory ligands such as PD-L1, thereby attenuating T-cell activity [41]. The glycolytic enzyme ENO1 (α-Enolase) was shown to upregulate PD-L1 in a HIF-1α-dependent manner, hampering CD8+ T cell infiltration and leading to PDAC progression [42]. Furthermore, PDAC tumors classified as more hypoxic by gene signatures display a more immunosuppressed TME and higher PD-L1 expression [1,11].

These converging mechanisms position hypoxia as a central architect of PDAC’s immune-cold, immune-excluded state and suggest that targeting hypoxia could help convert PDAC into an immune-hot tumor that is more amenable to immune checkpoint inhibition. In that respect, tools are required to identify the degree of tumor hypoxia as a surrogate marker for immunosuppression which could then inform the appropriate treatment path for successful immunotherapy.

## 3. Hypoxia Reprogramming of Cancer-Associated Fibroblasts and Its Impact on the Tumor Immune Microenvironment

Cancer-associated fibroblasts (CAFs) represent a major component of the TME in many cancer types. In PDAC, the highly prevalent CAF population in the TME promotes extensive fibrosis and desmoplasia, constituting a key driver of therapeutic resistance, recurrence, and metastasis. These cells share characteristics similar to fibroblasts activated following tissue injury or inflammation. The primary source of CAFs is thought to be the activation of resident quiescent fibroblasts in many tumor types. However, CAFs can also originate from a diverse range of other cell types, including endothelial cells, pericytes, adipocytes and mesenchymal stem cells [43]. In PDAC, resident quiescent pancreatic stellate cells (PSCs) are thought to be the primary source of CAFs [44]. Within the TME, the generation of CAFs is critically driven by various cytokines and growth factors released by cancer and infiltrating immune cells. Key regulatory molecules identified in this process include Transforming Growth Factor-β (TGF-β), Platelet-Derived Growth Factor (PDGF), Epidermal Growth Factor (EGF), Fibroblast Growth Factor (FGF), reactive oxygen species (ROS), Tumor Necrosis Factor (TNF), and Interleukin-1 β (IL-1 β) and -6 (IL-6) [45].

CAFs are characterized by the expression of standard fibroblast markers (for example: fibroblast-specific protein-1 (FSP-1/S100A4) or PDGF receptors (PDGFRs) α/β) alongside activation markers like fibroblast-activation protein (FAP), α-smooth muscle actin (α-SMA), periostin (POSTN), podoplanin (PDPN), tenascin-C (TNC) or caveolin-1 (CAV1). However, their precise identification is complicated because no single marker is CAF-specific, and their expression profiles vary widely, underscoring the significant heterogeneity within the TME. This variation has led to the categorization of CAFs into several subsets, including four main subsets in breast cancer (CAF-S1 to -S4), defined by the combined expression of markers such as FAP, CD29, and α-SMA [46]. Additionally, the CAF-S1 subset, which is associated with immunosuppression and inflammation and has also been identified in other tumors including PDAC [47], is further differentiated into two major functional types: inflammatory iCAFs (αSMA^LOW^), which are located away from the cancer cells and produce pro-inflammatory and immunomodulatory factors; and myofibroblastic myCAFs (αSMA^HIGH^), which reside close to the tumor and primarily secrete ECM components [48]. By directly interacting with tumor cells and secreting various factors (such as extracellular matrix (ECM), matrix metalloproteinases (MMPs), cytokines, chemokines, or vascularization-inducing proteins), CAFs exercise important regulatory functions within the TME. They also significantly promote tumor progression, metastasis formation, and interfere with the response to conventional therapies and the efficacy of antitumor immune responses [49,50], and are of particular importance in pancreatic cancer [51]. However, a contradictory study in mice also suggested that the depletion of αSMA+ CAFs, starting at either non-invasive precursor (pancreatic intraepithelial neoplasia) or the PDAC stage led to invasive, undifferentiated tumors with enhanced hypoxia, epithelial-to-mesenchymal transition, and cancer stem cells, with diminished animal survival [52].

Of note, the role of hypoxia in CAF reprogramming is central but contradictory. On one hand, hypoxia is pro-CAF: it drives tumor cells to release differentiation factors like TGF-β and PDGF, promotes fibroblast activation via ROS production, and upregulates activation markers such as FAP and α-SMA in CAFs in several tumor models including melanoma and hepatocellular carcinoma [53,54,55]. Similarly, hypoxia associated with chronic pancreatitis and PDAC also have salient effects on prolonged CAF activation [56]. However, other findings indicate that hypoxia can be inhibitory. For instance, studies using head and neck and vulval CAFs in 3D culture showed that hypoxia-induced HIF-1α stabilization (by inhibiting PHD2) led to the reversal of CAF activation [57].

These divergent results underline the probable existence of different hypoxic responses among CAF subsets and might also depend on the tumor type. Nevertheless, hypoxia profoundly influences CAF functionality within the TME, acting as a major driver of tumor aggressiveness, especially in PDAC. For instance, hypoxic CAFs drive metastasis by triggering the EMT in PDAC tumor cells [58]. Pancreatic CAFs stimulate the motility of pancreatic cancer cells through IGF1/IGF1R signaling under hypoxia [59]. Hypoxia also promotes CALB2+ CAF subset activation and the acquisition of an inflammatory phenotype, which promoted PDAC cell migration and patient-derived organoid growth in vitro and in vivo [60]. Furthermore, CAFs maintain critical pancreatic cancer cell lipid homeostasis upon oxygen deprivation [61]. Additionally, recent evidence confirms that hypoxia significantly enhances the immunomodulatory properties of CAFs in PDAC, generally promoting an immunosuppressive and protumor microenvironment. For instance, in mouse models, iCAFs are frequently found in hypoxic zones in PDAC, where their hypoxic signature amplifies the expression of inflammatory genes. This phenotype is so potent that HIF-1α stabilization alone can induce an iCAF phenotype and promote tumor growth in PDAC models [62,63]. Hypoxic CAFs also contribute to T cell dysfunction and poor prognosis by increasing the expression of enzymes like Arginase 2 (Arg2) in PDAC [64]. Interestingly, the CAF-specific deletion of HIF-2α, but not HIF-1α, significantly decreased the intratumoral recruitment of immunosuppressive M2 macrophages and Tregs in a PDAC mouse model [65]. Lastly, BNIP3 (BCL2/adenovirus E1B 19 kDa protein-interacting protein 3) + CAFs associated with hypoxia and inflammation predict immunotherapy response in PDAC [66].

In conclusion, and based on current studies, hypoxic stress is likely a key determinant in CAF generation and unequivocally enhances the CAF-dependent regulation of crucial protumor processes in PDAC, including ECM dynamics, cell metabolism, metastasis, immune response, and therapeutic resistance. Consequently, strategies targeting hypoxia within the TME, such as strategies targeting HIFs, may be an additional indirect way to target CAFs [67]. However, CAF heterogeneity might also represent a challenge, and whether hypoxia-targeting strategies might directly and effectively affect the several subtypes of CAF and counteract their pro-tumorigenic properties in PDAC, and in other tumor types, clearly requires further studies.

## 4. Hypoxia, EMT, and Cancer Stemness: Linking Tumor Plasticity to Immune Resistance

Hypoxia and HIF signaling are central drivers of tumor heterogeneity, promoting cellular phenotypes associated with cancer progression, metastasis, and therapeutic resistance. In hypoxic TME, HIF stabilization activates transcriptional programs that enhance cancer stem cell (CSC) phenotypes [68], increase metastatic potential [69], and confer resistance to chemotherapy [70] and radiotherapy [71].

Hypoxia exerts major selective pressure shaping tumor evolution. HIF signaling induces the expression of CSC markers, pluripotency-related transcription factors, and key signaling pathways that collectively expand CSC-like populations with high phenotypic plasticity and stress adaptability. This hypoxia-driven plasticity supports the emergence and maintenance of therapy-resistant tumor clones and contributes to disease relapse [72]. Stem-cell-associated cancer markers, particularly in pancreatic cancer, include both membrane bound and intracellular proteins whose increase in expression is linked to poorer clinical outcomes. These markers include Promonin 1 (*PROM1*/CD133), CD24, CD44, ABCB1 (ATP Binding Cassette Subfamily B Member 1), ABCG2 (ATP-binding cassette sub-family G member 2), SOX2 (SRY-box transcription factor 2), *POU5F1* (POU class 5 homeobox 1)/OCT4 (Octamer-binding transcription factor 4), NANOG, EpCAM (Epithelial Cell Adhesion Molecule), CXCR4 (C-X-C motif chemokine receptor 4) [73], CD9 [74] and CD47 [75]. Together, these markers reflect the expansion of stem-like tumor cell populations that contribute to tumor heterogeneity and therapeutic resistance.

In addition to promoting CSC expansion, hypoxia reinforces CSC survival by regulating surface markers that contribute to immune evasion. CD24, a highly glycosylated membrane protein and transcriptional target of HIF-1α [76], is frequently overexpressed in pancreatic cancer cells and promotes immune escape through its interaction with the inhibitory receptor Siglec-10 expressed on macrophages and NK-cells [77]. This interaction suppresses immune-mediated tumor clearance and promotes tumor progression. Accordingly, targeting CD24, by using anti-CD24 monoclonal antibodies, chimeric antigen receptor (CAR)-redirected anti-CD24 T-cells, or targeting its interaction with Siglec-10 or in combinatory therapy with other drugs, has had a positive impact in PDAC [77]. Similarly, CD47, which functions as an immune checkpoint molecule, correlates with advanced disease stage and poor prognosis [78]. Antibody-specific targeting of CD47 restores the macrophage-mediated phagocytic uptake of cancer cells and increases apoptosis in PDAC models [75]. Interestingly, cells surviving the CD47 blockade exhibit reduced expression of *PROM1*/CD133 and showed improved response to combination chemotherapy, suggesting that immune checkpoint targeting may also influence CSC populations [75].

A central signaling axis connecting hypoxia, EMT, and immune regulation in pancreatic cancer involves the transcription factor STAT3 (Signal Transducer and Activator of Transcription 3) and its downstream effectors. The phosphorylation of STAT3 at tyrosine 705 (Y705) induces its dimerization and nuclear translocation, activating the transcription of genes involved in cell proliferation and cell survival [79]. The inhibition of STAT3 phosphorylation suppresses tumor growth and metastasis in PDAC [80]. STAT3 signaling also shapes the immune microenvironment as Y705 phosphorylation has been associated with an immune-cold TME in PDAC [81] and increased PD-L1 expression in natural killer/T-cell lymphoma [82]. One key regulator of the STAT3 signaling pathway is the transcription factor SOX9 [83] which is frequently overexpressed in pancreatic cancer and correlates with poor prognosis and therapy resistance [84]. Hypoxic signals induce SOX9 through both HIF-1α and HIF-2α, while SOX9 can enhance HIF-1α transcriptional activity by forming a positive feedback loop that amplifies hypoxia signaling [85]. Beyond tumor cells, SOX9 shapes stromal remodeling within the TME. Activated myofibroblasts increase collagen production, facilitating tumor metastasis. However, paradoxically, inhibiting these fibroblasts in mouse models accelerated PDAC progression and reduced survival [86]. Mechanistically, the deletion of collagen I from activated myofibroblasts induces a SOX9-dependent upregulation of CXCL5, promoting the recruitment of MDSCs and suppression of CD8^+^ T cell activity [86]. Finally, TWIST1 (twist family bHLH transcription factor 1), a target gene of both STAT3 and HIF signaling [87], further links hypoxia signaling to metabolic and immune regulation. In pancreatic cancer TWIST promotes glycolysis and the Warburg effect [88]. The knockdown of TWIST1 significantly reduces the expression of the immune checkpoint protein VISTA (V-domain Ig suppressor of T cell activation) in pancreatic cancer cells, an effect enhanced by cotreatment with the histone deacetylase inhibitor Vorinostat [89].

Overall, hypoxia-driven interactions between cancer cells and stromal and immune cells reshape the TME and contribute to plasticity and heterogeneity. However, hypoxia-induced effects are highly heterogeneous and can vary depending on the duration, intensity, and spatial distribution of oxygen deprivation within the TME. Moreover, the reliance on canonical CSC markers may oversimplify the dynamic and context-dependent nature of stem-like states. These dynamic conditions make effective targeting challenging but also intellectually compelling. Successful therapies will likely require approaches that simultaneously target malignant cells, modulate the extracellular matrix and enhance antitumor immune responses alongside continuous monitoring, to achieve durable clinical benefit in pancreatic cancer.

## 5. Hypoxia-Driven Oxidative Stress and Redox Adaptation Impedes Immune Response

Despite reduced oxygen availability, hypoxic regions within pancreatic tumors paradoxically exhibit elevated reactive oxygen species (ROS) production, creating a complex interplay between oxidative stress and hypoxic adaptation [90,91,92,93]. Under hypoxic conditions, mitochondrial ROS generation increases as electrons leak from the disrupted electron transport chain, and these ROS stabilize HIF-1α by inhibiting PHDs [90,91]. This creates a positive feedback loop in which hypoxia-induced ROS fuels hypoxic signaling, while the resulting HIF-1α stabilization further shapes the metabolic and immunosuppressive landscape of the TME.

ROS exerts context-dependent effects that shift between pro-survival signaling and cytotoxic damage. At moderate concentrations, ROS serve as secondary messengers that activate proliferative and pro-survival pathways including PI3K/AKT, ERK1/2, NF-κB, and HIF-1α, thereby promoting cell proliferation, migration, EMT, and resistance to chemotherapy and radiotherapy [94,95,96,97]. Conversely, when ROS levels exceed a critical threshold, they trigger cell death pathways mediated by p53, JNK, p38, and ATM, among other pathways [96,97]. Overall, a tightly controlled oxidative environment is required to confer a selective growth and survival advantage while avoiding oxidative catastrophe.

In that respect, pancreatic cancer cells respond to pro-oxidative stress arising from uncontrolled proliferation and limited vascular supply by upregulating antioxidant defenses (reviewed in [92]). The redox signaling protein apurinic/apyrimidinic endonuclease 1/Redox effector factor 1 (APE1/Ref-1) regulates redox homeostasis in pancreatic cancer cells and contributes to cell proliferation and migration through NF-κB (nuclear factor kappa-light-chain-enhancer of activated B cells) and HIF-1 pathways [98]. Central to this adaptive response is the NRF2 (nuclear factor erythroid-related factor 2) transcription factor, which orchestrates the expression of cytoprotective genes, including the glutathione system, in response to oxidative and environmental stressors [6,92,96]. The KRAS-NRF2 axis is particularly central in PDAC, as mutant KRAS drives chronic ROS production that activates NRF2 not only to coordinate antioxidant responses but also to orchestrate broader metabolic reprogramming, including the regulation of inducible nitric oxide synthase (NOS2) and the modulation of reactive nitrogen species [93,99,100]. Chronic hypoxia enhances glutathione-dependent antioxidant capacity, protecting cell membranes from concomitant oxidative damage, with glutathione peroxidase maintaining redox balance in Panc-1 pancreatic tumor cells [101]. Similarly, the NRF2-regulated enzyme heme oxygenase-1 confers a survival advantage to PDAC cells under hypoxia, wherein its inhibition increases ROS production and cell death [102].

The critical role of antioxidant enzymes in pancreatic cancer cell survival under hypoxia-induced ROS is further exemplified by the activity of superoxide dismutases (SODs). Under hypoxia, reduced SOD1 expression decreases the viability of PANC-1 and MiaPaCa-2 PDAC cells, while SOD2 shields KP4 pancreatic carcinoma cells from hypoxia/reoxygenation-induced oxidative stress (reviewed in [92]). In addition, pancreatic stellate cells (PSCs) also experience oxidative stress under hypoxia and adapt by upregulating SOD1 and SOD2 expression alongside enhanced NRF2 phosphorylation [103]. Furthermore, hypoxia-induced ROS stabilize HIF-1α and upregulate GLI1 in PSCs, triggering the secretion of pro-tumorigenic cytokines such as IL-6, SDF-1, and VEGF-A, which collectively promote invasion, ECM deposition, angiogenesis, and further immune exclusion [104]. This creates a self-amplifying cycle wherein hypoxia drives ROS production, ROS activate stromal cells, and desmoplastic matrix worsens vascular dysfunction and hypoxia.

Beyond stromal reprogramming, elevated ROS in the hypoxic TME directly impair effector immune cell function, suppressing CD8^+^ T-cell activation and cytotoxicity while promoting M2 macrophage polarization and MDSC immunosuppressive activity [105,106]. Meanwhile, the cancer cells’ robust antioxidant machinery protects them from oxidative damage, creating a selective survival advantage over immune effector cells and an oxidative gradient that reinforces immune exclusion and limits immunotherapy efficacy.

Both tumor and stromal cells’ reliance on redox adaptation presents a therapeutic vulnerability [92,96]. Targeting the NRF2-mediated antioxidant response can disrupt adaptive redox homeostasis and resensitize tumors to chemotherapy [99,107], while ROS-inducing agents may push cells beyond the survival threshold into apoptosis [108]. Conversely, combining antioxidant inhibition with strategies that alleviate hypoxia, such as vascular normalization or oxygen delivery agents, could collapse the hypoxia–ROS feedback loop and restore sensitivity to cytotoxic and immune-based therapies.

Importantly, ROS are generated from multiple subcellular compartments, including mitochondria (the primary source under hypoxia), and NADPH oxidases at the plasma membrane, the endoplasmic reticulum, and peroxisomes, each contributing distinct functional roles [97,109,110]. Accurate characterization of ROS dynamics across tumor compartments, accounting for spatial heterogeneity, temporal fluctuations, and technical limitations of detection methods, will be critical for translating redox-targeted strategies into effective clinical interventions (reviewed in [110]).

## 6. The Multifaceted Approaches Used to Target Hypoxia

Persistent oxygen deprivation induces adaptive signaling that supports angiogenesis, immune evasion, metabolic reprogramming, and resistance to therapy [4,111,112,113]. Central to this adaptation is the HIF pathway, a transcriptional hub that organizes cellular survival under reduced oxygen availability. While both HIF-1α and HIF-2α contribute to tumor progression, HIF-1α shows broader tissue expression and a more universal role across cancer types [8,9,10]. HIF-1α overexpression is associated with increased patient mortality in multiple solid tumors, including PDAC, where it drives cancer stem cell specification, chemoresistance, and metastatic dissemination [3,9,114]. By contrast, HIF-2α function is more tissue-specific, with predominant roles in clear cell renal cell carcinoma (ccRCC) and certain other malignancies [115,116,117]. This distinction has important therapeutic implications: while HIF-2–selective inhibitors such as Belzutifan have proven effective in VHL-deficient ccRCC, dual HIF-1/HIF-2 targeting is likely required for broader applicability, particularly in the framework of immunotherapy [9,112]. Therapeutic strategies aimed at hypoxia can be grouped into: (i) drugs that directly target HIFs, (ii) hypoxia-activated prodrugs, (iii) vascular and oxygen delivery approaches (including nanomedicine), and (iv) rational combinations with immunotherapy, with specific considerations in PDAC.

### 6.1. Targeting the HIF Pathway: HIF-1α and HIF-2α as Complementary Targets

#### 6.1.1. Rationale for Dual HIF-1α and HIF-2α Targeting

The clinical success of HIF-2α inhibitors such as PT2385 and belzutifan in RCC has validated HIF-2α as a druggable target [115,116,117]. However, the broader tissue expression and fundamental role of HIF-1α in hypoxic adaptation underscore its importance as a therapeutic target across many solid tumors [8,10,112]. In PDAC, HIF-1α is frequently stabilized by a combination of severe hypoxia and oncogenic signaling (for example: KRAS, PI3K/AKT, and RAS/MAPK), and its expression correlates with poor prognosis and treatment failure [3,9,114]. Therefore, comprehensive hypoxia-directed therapy should address both HIF isoforms, with a particular emphasis on HIF-1α in pancreas-specific settings.

#### 6.1.2. Inhibitors of HIF Expression, Translation, and Stability

Small-molecule and nucleic-acid-based inhibitors can interfere with HIF biology at multiple levels. Antisense oligonucleotides such as EZN-2968 reduce HIF-1α mRNA expression and inhibit tumor growth in preclinical models [118]. Topoisomerase inhibitors (for example: topotecan) and receptor tyrosine kinase inhibitors suppress HIF-1α translation by disrupting the PI3K/AKT and MAPK signaling pathways that regulate protein synthesis [112,119]. Hsp90 inhibitors (for example: 17-AAG) promote HIF-1α degradation via VHL-independent mechanisms [120]. Echinomycin blocks HIF-1 binding to HREs, decreasing the transcription of *VEGF* (Vascular Endothelial Growth Factor) and *GLUT1* (Glucose transporter 1) [121]. Direct targeting of HIF dimerization and transcriptional activity has also advanced. Acriflavine, which disrupts HIF-1α/HIF-1β dimerization, reduces VEGF expression and tumor vascularization in preclinical pancreatic cancer models [9,114]. HIF-2α-selective antagonists such as PT2385 and Belzutifan bind the PAS-B domain of HIF-2α, preventing heterodimerization with ARNT and transcriptional activation [115,116,117]. Recently, proteolysis-targeting chimeras (PROTACs) have been developed to induce the targeted degradation of HIF-1α and/or HIF-2α, offering a means to more completely extinguish HIF signaling [10,122]. In PDAC, the dense desmoplastic stroma and profound hypoxia impede drug penetration. Nevertheless, preclinical blockade of HIF-1α has shown the partial reversal of desmoplastic signaling, improved perfusion, and enhanced chemosensitivity [3,114]. Given the dominance of HIF-1α in pancreatic tumors, HIF-1-directed strategies may offer greater benefit than HIF-2-selective approaches alone in this context.

#### 6.1.3. Dual HIF-1α and HIF-2α Inhibition with 32-134D

Small-molecule HIF inhibitors that simultaneously target HIF-1α and HIF-2α offer a complementary strategy to isoform-selective agents. In hypoxic hepatocellular carcinoma (HCC) cells, the low molecular weight 32-134D significantly reduces HIF-1α and HIF-2α protein accumulation, impairs the recruitment of HIF complexes and coactivator p300 to hypoxia-responsive elements, and suppresses a large fraction of the hypoxia-induced transcriptome, including genes controlling angiogenesis, metabolism, and immune regulation [123]. In human HCC xenografts, treatment with 32-134D results in the loss of intratumoral HIF-1α and HIF-2α protein expression, dose-dependent inhibition of tumor growth, and reduced vascularization, accompanied by the decreased expression of *VEGFA*, *ANGPTL4* (angiopoietin like 4), *EPO* (erythropoietin) and other HIF target genes that sustain angiogenesis and an immunosuppressive TME. Furthermore, 32-134D downregulates multiple immune checkpoints and adenosinergic mediators (including CD73, PD-L1, CD47, B7-H4, TIM-3, LDHA, CD39 and CA9 (carbonic anhydrase 9)) while increasing CXCL9 and CXCL10, thereby enhancing the recruitment of T cells and NK cells in syngeneic HCC models [123]. In tumor studies, 32-134D monotherapy inhibits HCC growth in immunocompetent mice and, when combined with anti-PD1 antibody, increases the rate of tumor eradication without inducing anemia or apparent organ toxicity [123]. These findings highlight how dual HIF-1/HIF-2 inhibition simultaneously blunts angiogenic, metabolic and immune-evasive programs and provides a strong mechanistic rationale for combining systemic HIF blockade with immune checkpoint inhibition. A key question is whether similar immune reprogramming can be achieved in PDAC, where stromal architecture and perfusion constraints may require the optimization of delivery, dosing, and combination timing.

#### 6.1.4. Targeting HIF-Regulated Surface Proteins and Downstream Pathways

Indirect targeting of the HIF program via HIF-regulated surface molecules and downstream pathways can attenuate hypoxia-driven tumor progression and immune escape. VEGF inhibitors such as bevacizumab and multi-targeted receptor tyrosine kinase inhibitors (for example: sunitinib and sorafenib) block HIF-induced angiogenesis and have demonstrated clinical benefit in several tumor types [124,125]. HIF-1α drives the expression of CA9, CD39/CD73, and other pH- and adenosine-regulating enzymes that contribute to an acidic, adenosine-rich, immune-cold microenvironment [10,126]. CA9-directed antibodies (for example: girentuximab) and CD73 blockade are in clinical or preclinical development and have demonstrated synergy with chemotherapy and checkpoint inhibitors in various models, including pancreatic cancer [127,128].

### 6.2. Alternative Hypoxia-Targeting Strategies

#### 6.2.1. Hypoxia-Activated Prodrugs (HAPs)

Hypoxia-activated prodrugs exploit low oxygen tension to selectively release cytotoxic species in hypoxic tumor regions. Agents such as tirapazamine (TPZ), evofosfamide (TH-302), and PR-104 undergo bioreductive activation under hypoxia, preferentially killing poorly oxygenated tumor cells [113,129]. While TPZ showed compelling preclinical activity, randomized clinical trials combining TPZ with chemotherapy or radiotherapy yielded mixed results, in part due to a lack of robust hypoxia-based patient selection [113]. Evofosfamide progressed further in PDAC. In early-phase studies, gemcitabine plus TH-302 showed encouraging activity [130,131]. However, the phase III MAESTRO trial in advanced pancreatic adenocarcinoma failed to meet its primary overall survival endpoint when evofosfamide was added to gemcitabine [132]. These setbacks underscore the need for validated imaging or gene-expression hypoxia biomarkers and improved spatial–temporal characterization of hypoxia to identify patients most likely to benefit [133]. Mathematical models and preclinical studies suggest that optimizing dosing schedules and integrating hypoxia imaging may help unlock the potential of HAPs [129,134].

#### 6.2.2. Vascular Normalization and Oxygen Delivery Strategies

Anti-angiogenic therapy transiently normalizes tumor vasculature, improving perfusion and facilitating drug and immune cell access [125,135,136]. The ‘vascular normalization window’ may be most beneficial when combining VEGF-targeted agents with radiotherapy or immunotherapy [124]. In parallel, interventions that alleviate acidosis (for example: CA9 inhibition) can improve immune cell function within the hypoxic niche [126]. In PDAC, the combination of dense stroma, poor perfusion, and severe hypoxia is a central therapeutic barrier. Myo-inositol trispyrophosphate (ITPP) has emerged as a non-toxic oxygen-modulating compound that mechanistically differs from existing hypoxia-targeting agents. Anti-angiogenic therapies restrict new vessel formation but can worsen hypoxia by over-pruning the vasculature [137]. ITPP acts as an allosteric effector of hemoglobin, enhancing oxygen release preferentially in hypoxic tissues while avoiding systemic hyperoxia [138]. The restored oxygen levels reactivate PHDs, triggering HIF-1α and HIF-2α degradation [139,140]. Consequently, the entire hypoxic signaling cascade collapses, reducing the transcription of genes that promote angiogenesis (*VEGFA*), glycolysis (*SLC2A1* and *LDHA*), extracellular matrix remodeling, and immune evasion. While this can also be achieved by HIF inhibitors, these act downstream, targeting individual nodes within a redundant network. ITPP, by contrast, directly reoxygenates the tumor and simultaneously induces functional vascular normalization, transforming chaotic, leaky tumor vessels into more organized, perfused networks [141]. This improves perfusion, reduces interstitial pressure, and facilitates both drug delivery and immune cell trafficking. Furthermore, ITPP significantly potentiates chemotherapy efficacy in multiple small-animal models of cancer [139,140,141,142,143], including PDAC, wherein it reduced hypoxia, decreased metastasis, and enhanced gemcitabine efficacy [139]. By alleviating hypoxia, ITPP offers a way to reprogram PDAC’s hostile microenvironment, as increased oxygen tension has been previously shown to restore effector T-cell metabolism and cytotoxicity, reduce regulatory T-cell recruitment, and shift macrophage polarization from M2 to M1 phenotypes [138,144,145,146]. Preclinical studies combining ITPP with PD-1 blockade showed additive antitumor effects [146]. Moreover, ITPP was found to be safe and well-tolerated in the first-in-patient Phase Ib dose-escalation study that enrolled patients with advanced hepato-pancreato-biliary cancers, including PDAC [147]. Therefore, integrating ITPP into immunotherapy protocols may convert ‘immune-cold’ PDAC into ‘immune-hot’ tumors, potentially unlocking durable immune control where current therapies fail. In that respect, hyperbaric oxygen therapy (HBOT) has similarly been studied as a means to increase tumor oxygenation; a Cochrane review and other clinical data suggest potential synergy with radiotherapy, although practical limitations and inconsistent trial designs have limited wider use [148,149]. Oxygenation-based approaches are attractive as they act upstream of multiple hypoxia programs; however, their clinical impact will depend on the reproducible delivery of oxygen modulation, appropriate scheduling with immunotherapy/chemotherapy, and confirmation that reoxygenation is achieved within PDAC’s poorly perfused tumor core.

#### 6.2.3. Nanomedicine-Driven Oxygen Delivery and Hypoxia Modulation

Nanomedicine offers opportunities to deliver oxygen, hypoxia-modulating agents, or HAPs selectively to the TME. Oxygen-supplied nanomaterials encapsulating oxygen carriers (perfluorocarbons and hemoglobin), catalase, or HAPs can both alleviate hypoxia and enhance local drug accumulation [150,151]. Such platforms enable the spatiotemporal control of oxygen delivery, for example, perfluorocarbon-loaded nanoparticles that release oxygen under near-infrared irradiation to enhance radiotherapy and elicit systemic antitumor immunity [150,151]. Although most data remain preclinical, these systems are particularly appealing for hypoxic, poorly perfused malignancies such as PDAC.

### 6.3. Synergizing Hypoxia Targeting with Immunotherapy

#### 6.3.1. HIF Inhibition Combined with Immune Checkpoint Blockade

Hypoxia promotes immune evasion by upregulating immune checkpoint molecules (for example: PD-L1 and CD47), recruiting immunosuppressive MDSCs and regulatory T cells (Tregs), and impairing CTL and NK cell function [41,152,153,154]. Preclinical work demonstrates that HIF inhibition can enhance anti-PD-1/PD-L1 efficacy by increasing T-cell infiltration, reducing MDSC accumulation, and reversing key features of the immunosuppressive TME [9,10,155]. In RCC models, HIF-2α antagonists such as PT2385 or Belzutifan augment PD-1/PD-L1 blockade, and ongoing clinical trials are testing combinations of HIF-2 inhibitors and checkpoint inhibitors in patients with advanced kidney cancer [115,116,117]. In PDAC, preclinical HIF-1α blockade in combination with PD-1/PD-L1 or CTLA-4 antibodies reduces Treg prevalence and restores CD8+ T-cell activity, suggesting that dual targeting of HIF-1α and checkpoint pathways may help convert this ‘cold’ tumor into an immunoresponsive disease [3,114,152]. Successful clinical translation will depend on treatment sequencing and biomarker-guided selection, as partial HIF blockade or incomplete delivery to hypoxic niches may be insufficient to relieve immune exclusion in PDAC.

#### 6.3.2. Metabolic and Adenosinergic Modulation

HIF-1α mediates a metabolic shift towards glycolysis, lactate production, and adenosine accumulation, all of which suppress antitumor immunity [119,126,156]. The upregulation of CD39 and CD73 under hypoxia generates extracellular adenosine, which signals via A2A receptors to impair effector T cells and NK cells while supporting Tregs and MDSCs [41,152]. Therapeutic strategies targeting these pathways, including glycolysis inhibitors, A2A receptor antagonists, and CD73-blocking antibodies, are being investigated as rational combinations for checkpoint blockade [127,155]. In preclinical models, dual CD73 and PD-1 blockade demonstrated synergistic activity in melanoma and pancreatic cancers, enhancing T-cell infiltration and effector function [127,152]. These combinations are designed to balance local immune reprogramming with systemic metabolic effects and will likely require careful patient selection and pharmacodynamic readouts to confirm target engagement in hypoxic PDAC regions.

#### 6.3.3. Hypoxia-Adaptive Engineered Immune Cells and Oncolytic Platforms

Engineering immune cells and oncolytic vectors to respond to hypoxia offers another strategy to exploit the hypoxic TME while limiting systemic toxicity. CAR-T cells equipped with hypoxia-responsive promoters can restrict effector activity to hypoxic tumor sites, increasing local potency while reducing on-target/off-tumor toxicity [157]. Similar oxygen-sensing regulatory elements have been incorporated into oncolytic viruses and cytokine-expressing constructs to achieve TME-restricted activation [157]. These approaches are attractive for hypoxic, anatomically constrained tumors such as PDAC, though they remain in early-stage development.

### 6.4. Current Limitations and Future Directions in Targeting Hypoxia Signaling in PDAC

Although hypoxia-directed strategies represent an attractive opportunity in PDAC, durable clinical benefit has remained difficult to achieve, reflecting several recurring limitations across approaches discussed above [6,96,129]. First, target selection may be complicated by biological redundancy within the HIF network, as well as by compensatory pathways that may sustain angiogenic, metabolic, and immune-evasive programs despite direct pathway inhibition. Second, systemic toxicities may constrain dosing and combinations, as many hypoxia and HIF-regulated processes are shared with normal physiology; as a result, on-target effects outside the tumor may limit the therapeutic window. Third, drug delivery remains a central barrier in PDAC; dense desmoplasia, poor and heterogeneous perfusion, and elevated interstitial pressure impede penetration into the most hypoxic regions, where these interventions are intended to act. Finally, hypoxia itself is spatially and temporally heterogeneous, and the absence of standardized, validated hypoxia biomarkers has limited patient stratification and the ability to confirm target engagement in early clinical studies.

Future progress is likely to benefit from biomarker-guided trial design and integrated solutions that address both biology and delivery [1,6,96,151]. Incorporating hypoxia assessment (discussed in Section 7) may enable enrichment for patients with a clear hypoxic, immune-excluded phenotype and support pharmacodynamic readouts of reoxygenation or HIF-pathway suppression. Combination strategies that pair hypoxia modulation with chemotherapy and immune checkpoint blockade may be particularly relevant in PDAC, provided that sequencing and scheduling account for transient windows of improved perfusion and immune access. Parallel advances in delivery, including nanomedicine platforms, stromal and vascular normalization approaches, and hypoxia-adaptive cellular or viral systems, may enhance penetration into hypoxic niches while limiting systemic exposure. Together, these directions support a precision framework in which the distribution and dynamics of hypoxia guide the selection of patients, the choice of hypoxia-targeting modality, and rational combination regimens.

## 7. Hypoxia Biomarkers with Clinical Translational Potential

Despite robust biological rationale, translating hypoxia-targeting strategies into consistent clinical benefit has been challenging. The success of such strategies depends on the availability of biomarkers to identify patients that are most likely to benefit from such interventions. Twelve clinical trials in pancreatic cancer patients explored hypoxia detection methods. These methods included: immunohistochemical (IHC) staining of endogenous hypoxia markers (HIF1α or downstream factors (NCT01995240 and NCT03718650)), or exogenous hypoxia tracers (pentafluoroethyl-nitroimidazole (EF5) and pimonidazole (NCT00087191, NCT01248637, and NCT03718650)); positron emission tomography (PET) imaging of radiolabeled hypoxia tracers, ^18^F-fluoromisonidazole (^18^F-FMISO), ^18^F-EF5, ^18^F-Fluoroazomycin arabinoside (^18^F-FAZA), and ^18^F-flortanidazole (^18^F-HX4) (NCT00047710, NCT01123005, NCT01542177, NCT01995084, NCT01989000, NCT02496832, NCT03168737, and NCT04395469); as well as functional magnetic resonance imaging (MRI) (NCT01995240 and NCT01989000) (Table 1).

Immunohistochemical (IHC) staining of HIF-1α and its downstream targets has been incorporated in clinical trials as a method for validating hypoxia [167,168]. The issue with such markers is that their expression is not exclusively regulated by hypoxia and they cannot be solely relied upon to represent the tumor’s hypoxic state. On the other hand, exogenous hypoxia-sensing agents, the 2-nitroimidazole derivatives EF5 and pimonidazole, get reduced at low oxygen, forming covalent adducts with cellular macromolecules only in hypoxia. Such agents, however, are intravenously injected into patients and require subsequent detection by IHC of a tumor biopsy. While the outcome of a clinical trial on pimonidazole (NCT01248637) suggested that it would be feasible to stratify patients based on their tumor’s hypoxic state (Table 1), markers that require detection by IHC are still prone to the disadvantages of this technique (Table 2).

The heterogeneous nature of hypoxia in PDAC suggests that functional imaging could be the preferred method to monitor and report on this condition. In that respect an alternative approach was the application of radiolabeled tracers, ^18^F-FMISO, ^18^F-EF5, ^18^F-FAZA, and ^18^F-HX4, which are reduced in hypoxic cells and can be evaluated by PET imaging. The results from NCT01542177 on ^18^F-FAZA show that while PDAC can be highly hypoxic, it is not the case for all patient tumors, reinforcing the relevance of hypoxia evaluation in informing hypoxia-targeting strategies [160]. The next-generation radiotracer, ^18^F-HX4, was the subject of two clinical trials (NCT01995084 and NCT01989000) which showed encouraging findings (Table 1). Unfortunately, no further studies were conducted to determine its utility in predicting response to hypoxia-targeted therapy. Functional MRI was additionally investigated as a non-invasive technique to report tumor oxygenation and profusion (Table 1); however, the inherent limitations of imaging techniques including the variability in image acquisition and processing as well as the absence of fully standardized imaging parameters has limited their extrapolation to large-scale applications.

Every technique has its own advantages and limitations (Table 2) and there is yet to be a validated hypoxia biomarker that is being implemented in the clinical setting to predict response to hypoxia-targeted therapy in PDAC.

### Gene Signatures as an Emerging Hypoxia Biomarker

Known targets of HIF1α, such as *LDHA* [169], have shown individual prognostic potential in pancreatic cancer. A single gene is incapable of robustly representing the complex and heterogeneous nature of a tumor’s hypoxic state. Hypoxia gene signatures composed of multiple genes could capture different facets of the TME providing a more representative and clinically relevant picture of hypoxia.

Most signatures to date are bioinformatic signatures that relied on probing hypoxia-related gene sets in PDAC patient datasets followed by the derivation of a prognostic model based on the genes associated with survival (reviewed in [1,170]). One caveat of such an approach is the biological relevance of the selected genes in hypoxic PDAC. This has been at least partially addressed in only four signatures [11,171,172,173] (Table 3).

Among the signatures, two were bioinformatic, derived following a similar approach from the same dataset (Table 3). The in vitro validation of the genes was done in a single PDAC cell line, and the exact hypoxic conditions (pO_2_) and incubation time were not reported [171,172]. The association of the signatures with survival was evaluated in two PDAC cohorts, the second cohort having very few cases, and neither signature was validated as an independent prognostic factor. Furthermore, their performance was not compared to any other hypoxia gene signature.

The Buffa 51-gene hypoxia signature has shown remarkable prognostic power in multiple tumor types [173]. It was recently applied in a single PDAC cohort and shown to be an independent prognostic factor [174] (Table 3). Five genes among this metagene signature were found to be predictive of the hypoxic state of tumors. Nonetheless, only one gene was examined in vitro and shown to be significantly upregulated in one PDAC cell line upon hypoxia exposure.

On the other hand, the 8-gene hypoxia signature which was derived from a list of 15 hypoxia-related genes, was validated in a panel of cell lines [11] (Table 3). The signature acted as an independent prognostic factor in two PDAC datasets, was predictive of survival, and outperformed the only other published PDAC hypoxia gene signature at the time [11]. Such features make this signature a top contender for clinical validation.

Of relevance, the stratification of patients based on tumor hypoxia enables the comparison of other tumor features including those implicated in response to immunotherapy [11,171,172,174]. Reported correlations between hypoxia signatures and the immune landscape of PDAC underscore the immunosuppressive nature of more hypoxic tumors (Table 3). This further supports hypoxia alleviation to enhance immunotherapy response and the relevance of hypoxia gene signatures in identifying potential responders.

The success of hypoxia gene signatures in other cancers and their implementation in clinical trials (reviewed in [175]) highlights the need to validate and apply a PDAC hypoxia gene signature. Regarding the path of such a signature to the clinic, this awaits a few critical steps [1,175,176]. First, how well a signature reflects tumor oxygenation needs to be validated. Second, a compatible assay needs to be developed by probing IHC tissue blocks or tumor biopsies, which are more representative of available patient tissue material. Third, a proper cut-off needs to be identified for stratifying patient tumors, and this along with the signature’s prognostic and predictive power must pass prospective validation.

## 8. Conclusions

Within the TME, hypoxia orchestrates metabolic, vascular, stromal, and immune alterations that drive defects in innate and adaptive antitumor immunity. These changes foster immune evasion, restrict drug delivery, and facilitate tumor progression, thereby positioning hypoxia as a major barrier to both chemotherapy and immunotherapy, including ICIs.

A broad array of hypoxia-directed interventions has emerged, including HIF-1α/HIF-2α inhibitors, hypoxia-activated prodrugs, vascular normalization and oxygen delivery strategies, as well as hypoxia-adapted cellular and oncolytic platforms. While HIF-2α inhibitors have validated the HIF pathway as a druggable axis in clear cell renal cell carcinoma, the wider expression and foundational role of HIF-1α in many solid tumors underscore the need for approaches that target both isoforms, particularly in hypoxic, stroma-rich malignancies such as PDAC. Agents that alleviate hypoxia at its source, such as the allosteric hemoglobin effector ITPP, are particularly attractive because they simultaneously reoxygenate tumors, dampen HIF-1α/2α signaling, normalize aberrant vasculature, and resensitize cancers to cytotoxic therapies and ICIs.

A persistent obstacle to clinical translation of hypoxia-targeted strategies in PDAC is the lack of robust hypoxia biomarkers capable of capturing the spatial and temporal heterogeneity of tumor oxygenation and predicting benefit from hypoxia-targeted therapies. Functional imaging, exogenous tracers, and immunohistochemistry have provided important insights but are limited by technical variability, whereas hypoxia gene signatures offer a promising route to stratify patients, refine prognosis, and link hypoxic burden to immune features of the TME.

Integrating hypoxia alleviation with immune checkpoint blockade, guided by dynamic hypoxia biomarkers and PDAC-specific gene signatures, may convert an immune-cold, hypoxia-adapted tumor into a more immune-responsive disease and transform hypoxia from a fixed liability into a modifiable determinant of treatment success.

## Figures and Tables

**Table 1:** Clinical trials including hypoxia detection in pancreatic cancer patients.

sn	Trial	Study Start	Study Type-Phase	Status	Cohort	Hypoxia Detection	Outcome
1	NCT00047710	2002	Interventional-Phase 1	Completed	Locally advanced pancreatic cancer	Gallium-68-labeled nitroimidazole derivative-PET	No results posted—last update 01-08-2012
2	NCT00087191	2004	Interventional-NA	Terminated	Abdominal or non-small cell lung cancer	Immunodetection of EF5 adducts	No results posted—last update 16-01-2013
3	NCT01123005	2010	Interventional-Phase 1	Terminated	Solid tumors	^18^F-EF5-PET	No results posted—last update 06-11-2017
4	NCT01248637	2010	Observational	Completed	Pancreatic cancer	Immunodetection of pimonidazole adducts	Pimonidazole staining was conducted on up to 10 FFPE tumor sections per patient followed by quantitative image analysis [158,159]. Ten patients undergoing surgical resection of localized pancreatic cancer were included. The reported tumor hypoxic percentage showed intra- and intertumoral heterogeneity. Sampling enough sections, the variance in the same patient’s tumor was < that across patients’ tumors [158,159,160].
5	NCT01542177	2012	Observational	Completed	Pancreatic cancer	18^F^-FAZA-PET	^18^F-FAZA PET conducted in 11 pancreatic cancer patients to determine intra- and intertumor heterogeneity of hypoxia [160]. The hypoxic percentage varied from 0 to 60%, with five patients demonstrating minimal hypoxia (<10%). A later study on 15 previously untreated PDAC patients found 75% of patients to have some degree of hypoxia [161]. The hypoxic fraction ranged from <5% to >50% and was not associated with tumor perfusion or volume.
6	NCT01995084	2012	Interventional-NA	Completed	Pancreatic and esophageal cancer	18^F^-HX4-PET	The optimal time point for imaging hypoxia and the reproducibility of measures accrued using ^18^F-HX4 was determined in 32 recruited patients, among whom 13 had PDAC [162]. Good reproducibility between two PET/CT scans taken on two separate days and imaged 3.5 h post injection of ^18^F-HX4 was reported.
7	NCT01989000	2013	Interventional-NA	Completed	Borderline resectable pancreatic cancer ^a^	Tumor cellularity and extracellular matrix composition with DWI-MRI, tumor vascularity by DCE-MRI; tumor hypoxia by T2* MRI and ^18^F-HX4 PET-CT	Different parameters from each MRI method were derived in 30 patients and correlated with immunohistochemical staining of HIF1α (hypoxia marker), VWF (vasculature marker), and PSR (collagen marker) in 15 patients with available histopathological data [163]. R2* (the reciprocal of T2* relaxation time) was the only parameter significantly associated with the amount of HIF1α nuclear staining. 18F-HX4-PET imaging outcomes in untreated patients (42 patients with locally advanced or metastatic PDAC (NCT01995084) or borderline resectable pancreatic cancer (NCT01989000)), were investigated and correlated with metastasis, survival and HIF1α immunohistochemical staining [164]. Patients with localized disease had significantly lower HX4 uptake compared to those with metastasized disease and OS was significantly shorter in the group with high tracer uptake. A good correlation between the degree of HIF1α staining in the 13 patients with available histopathology and HX4 uptake was reported [164].
8	NCT01995240	2013	Interventional-NA	Completed	Locally advanced or metastatic PDAC	DCE-MRI, T2* MRI and DWI to be compared with IHC markers of hypoxia among others and correlated with treatment outcome	T2*-weighted and DCE-MRI parameters have good repeatability [165]. Changes in patient tumors’ DWI models post-treatment could be distinguishable from variation observed in the absence of treatment [166].
9	NCT02496832	2014	Interventional-NA	Withdrawn (Study was never initiated)	Advanced pancreatic cancer	^18^F-FAZA-PET	No results posted—last update 19-02-2018
10	NCT03168737	2017	Interventional-Phase 1	Active, not recruiting	Malignant solid neoplasms	^18^F-FAZA-PET-CT	No results posted—last update 23-07-2025
11	NCT03718650	2021	Interventional-Early Phase 1	Withdrawn (Lack of funding)	Resectable pancreatic cancer	Staining for pimonidazole-Immunohistochemistry GLUT1 and CA-IX	No results posted—last update 08-02-2021
12	NCT04395469	2021	Interventional-NA	Active, not recruiting	Unresectable, non-metastatic, locally advanced unresectable pancreatic adenocarcinoma	^18^F-FAZA-PET-MRI	No results posted—last update 18-06-2024

**^a^** Defined based on the Dutch Pancreatic Cancer Group. CT: computed tomography; DCE: dynamic contrast enhanced; DWI: diffusion weighted imaging; EF5: pentafluoroethyl-nitroimidazole; FAZA: fluoroazomycin arabinoside; FFPE: formalin fixed paraffin embedded; HIF1α: hypoxia inducible factor 1α; HX4: flortanidazole; MRI: magnetic resonance imaging; NA: not applicable; OS: overall survival; PDAC: pancreatic ductal adenocarcinoma; PET: positron emission tomography; PSR: Picrosirius Red; VWF: von Willebrand factor.

**Table 2:** Advantages and disadvantages of hypoxia detection methods adapted in pancreatic cancer.

Technique	Advantages	Disadvantages
Oxygen electrode probes	Direct oxygen measurement	Invasive
	Well validated	Tissue damage by probe
		Tumor accessibility
IHC (Endogenous hypoxia marker (HIF1α, CA9, GLUT1) or Exogenous hypoxia tracer (EF5, pimonidazole))	Conducted on diagnostic biopsy	Unable to quantify pO_2_
	Simple to perform	Unable to assess dynamic changes
	Cheap	High sampling bias
		Low robustness
		Subject to interobserver bias
		Endogenous marker expression not specific to hypoxia, modified by factors
		Exogenous tracer to be administered prior to biopsy
		Exogenous tracer detects only severe hypoxia
PET (Exogenous radiotracer)	Dynamic changes can be assessed	Unable to quantify pO_2_
	Whole tumor analysis	Radiotracer to be administered prior to imaging
		Complex image analysis
		Limited resolution
		Expensive
MRI (R2*, DCE, DW)	Dynamic changes can be assessed	Unable to quantify pO_2_
	Whole tumor analysis	Absence of standardized and validated parameters
		Complex image analysis
		DCE MRI requires administration of contrast agent
		Expensive
Gene signatures	Conducted on diagnostic biopsy	Unable to quantify pO_2_
	Multiple genes increase robustness and replicability of results	Unable to assess dynamic changes
		No prospectively validated signature in pancreatic cancer

DCE (dynamic contrast enhanced): quantitative estimates of permeability; DW (diffusion weighted): indirect correlation with oxygen consumption based on cell density; IHC: immunohistochemistry; MRI: magnetic resonance imaging; PET: positron emission tomography; R2*: mapping of oxygen saturation level.

**Table 3:** Select prognostic hypoxia gene signatures in pancreatic cancer.

Sig.	Derivation	Scoring	Cohort	Group (number)	Survival ^a^			Immune ^b^			Ref.
					End Point	Univariate Cox PH/KM	Multivariate Cox PH	Method	Higher in Hypoxia-high/High-Risk Group	Higher in Hypoxia-Low/Low-Risk Group	
8-gene (*DDIT4*, *LDHA*, *MXI1*, *NDRG1*, *P4HA1*, *PGK1*, *SLC2A1*, *VEGFA*)	398 genes from published hypoxia prognostic or predictive signatures -> 15 genes based on frequency and biological relevance -> 8 genes based on ≥ 2-fold upregulation in hypoxia (1% O_2_) in a panel of cancer cell lines	Expression of each gene converted to a gene score of 1 or −1 depending on whether the expression is greater or less than the median expression in the entire cohort -> Hypoxia score (HS) calculated as the sum of gene scores -> Classification into hypoxia-high (HS > 0)/hypoxia-low (HS ≤ 0)	PAAD TCGA	High (66) vs. Low (98)	OS DSS PFS	1.9 (1.2–2.9) *p* = 0.004 2 (1.2–3.2) *p* = 0.005 1.7 (1.1–2.5) *p* = 0.011	1.7 (1.10–2.7) *p* = 0.016 1.6 (0.99–2.6) *p* = 0.056 1.5 (0.97–2.2) *p* = 0.067	22 immune cells using CIBERSORTx	M0 macrophages	CD8+ T cells	[11]
								Immune score		Immune score	
								Cytolytic index		Cytolytic index	
								4-chemokine signature		Chemokine score	
			E-MTAB-6134	High (136) vs. Low (173)	OS DFS	2.1 (1.6–2.8) *p* < 0.001 1.8 (1.3–2.3) *p* < 0.001	2.19 (1.6–3.0) *p* < 0.001 1.8 (1.39–2.5) *p* < 0.001	PD-L1 protein abundance	PD-L1		
3-gene *(CAPN2*, *PLAU*, *CCNA2)*	Enrichment analysis of 9211 DEGs in hypoxia-related pathways using GO and KEGG gene sets -> 30 genes based on STRING protein analysis -> Univariate Cox regression analysis -> 3-gene prognostic model with LASSO	Multiplying expression of 3 genes with their corresponding lambda LASSO correlation coefficient to calculate risk score (0.007 × *CAPN2* + 0.163 × *PLAU* + 0.317 × *CCNA2*) -> Classification into high-/low-risk ^c^	PAAD TCGA	High-risk (89) vs. Low-risk (89)	OS	1.82 (1.2–2.76) *p* = 0.005	-	24 immune cell markers using ssGSEA from GSVA	Macrophages, Th1, NK CD56^bright^ cells, Th2	Th17, pDCs, eosinophils, Tfh cells	[171]
			GSE62452	High-risk (33) vs. Low-risk (32)	OS	3.09 (1.62–5.87) *p* < 0.001	-	Immune checkpoints/regulatory markers	*CD276*, *TNFSF4*, *CD70*, *TNFSF9*, *CD44*, *CD80*, *CD274*, *CD40*, *TNFRSF9*, *PDCD1LG2*, *LGALS9*, *CD86*, *HHLA2*, *HAVCR2*, *NRP1*, *TNFRSF18*, *TNFRSF4*, *IDO1*, *CD160*		
3-gene (*PLAU*, *SLC2A1*, *CA9*)	Enrichment analysis of 9211 DEGs in hypoxia-related pathways using GO and KEGG gene sets -> 20 genes selected based on STRING protein analysis -> Univariate Cox regression analysis -> 3-gene prognostic model with LASSO	Multiplying expression of 3 genes with their corresponding lambda LASSO correlation coefficient to calculate risk score (0.231 x *PLAU* + 0.029 × *SLC2A1* + 0.056 × *CA9*) -> Classification into high-/low-risk ^c^	PAAD TCGA	High-risk (89) vs. Low-risk (89)	OS	*p* = 0.008	-	24 immune cell markers using ssGSEA from GSVA	Macrophages, Th1, NK CD56^bright^ cells, Th2	Th17, pDCs, eosinophils, Tfh cells, T-cells, CD8 T-cells	[172]
			GSE62452	High-risk (33) vs. Low-risk (32)	OS	*p* = 0.018	-	Immune checkpoints/regulatory markers	*CD276*, *TNFSF4*, *CD70*, *TNFSF9*, *CD44*, *CD80*, *CD274*, *TNFRSF18*, *CD40*, *PDCD1LG2*, *HHLA2*, *TNFRSF4*, *TNFRSF9*, *HAVCR2*, *TNFRSF25*, *LGALS9*, *CD86*	*CD160*, *CD40LG*, *ADORA2A*	
51-gene (Buffa signature [173])	None: original metagene signature derived based on co-expression networks with validated hypoxia seed genes in multiple cancers [173]	Hypoxia scoring using rank-based, single-sample scoring method (singscore package) -> Bottom quartile: Hypoxia Low; Top quartile: Hypoxia High	TCGA PAAD	High (44) vs. Low (44)	OS PFS	*p* < 0.001 *p* = 0.001	OS: 1.52 (1.23–1.9) *p* < 0.001	8 immune cells using MCP-counter		Myeloid dendritic cells, NK cells, CD3^+^ and CD8+ T cells	[174]
								cDC1 activation score		cDC1 score	
								Immune checkpoints/regulatory markers	*D47*, *CD276*, *HLA-G, LGALS1*, *LGALS2*, *LGALS3*, *LGALS4*, *NT5E*, *PTGS2*	*ENTPD1* and *ARG*	

^a^ Univariate and multivariate Cox PH analysis with the hazard ratio, 95% confidence interval in brackets and *p*-value—alternatively *p*-value of Kaplan–Meier analysis is reported. ^b^ Reported immune cell fractions present in at least two datasets for 8-gene signature. Other signatures are tested in one single dataset. ^c^ Method of classification was not reported. cDC1: conventional dendritic cells subset 1; DEGs: differentially expressed genes; DFS: disease-free survival; DSS: disease-specific survival; GO: gene ontology; GSA: Genome Sequence Archive; GSVA: Gene Set Variation Analysis; KEGG: Kyoto Encyclopedia of Genes and Genomes; KM: Kaplan–Meier; LASSO: least absolute shrinkage and selection operator; MCP-counter: Microenvironment Cell Populations-counter; MSI: microsatellite instability; NK: natural killer; OS: overall survival; pDCs: plasmacytoid dendritic cells; PD-L1: programmed cell death protein-ligand 1; PFS: progression-free survival; PH: proportional hazard; Ref: reference; Sig.: signature; ssGSEA: single-sample Gene Set Enrichment Analysis; Tfh: T-follicular helper cells; Th: T helper; vs.: versus.

## Data Availability

No new data were created or analyzed in this study. Data sharing is not applicable to this article.

## Abbreviations

The following abbreviations are used in this manuscript:

18F-FAZA	18F-Fluoroazomycin arabinoside
18F-FMISO	18F-fluoromisonidazole
18F-HX4	18F-flortanidazole
ABCB1	ATP Binding Cassette Subfamily B Member 1
ABCG2	ATP-binding cassette sub-family G member 2
ADAM10	a disintegrin and metalloproteinase domain-containing protein 10
ANGPTL4	angiopoietin like 4
APE1/Ref-1	apurinic/apyrimidinic endonuclease 1/Redox effector factor 1
Arg2	Arginase 2
AXIN2	Axis Inhibition Protein 2
BNIP3	BCL2/adenovirus E1B 19 kDa protein-interacting protein 3
CA9	carbonic anhydrase 9
CAFs	cancer-associated fibroblasts
CAR	chimeric antigen receptor
CAV1	caveolin-1
CCL2	C-C Motif Chemokine Ligand 2
ccRCC	clear cell renal cell carcinoma
cDC1	conventional dendritic cells subset 1
CSC	cancer stem cell
CTLA-4	cytotoxic T-lymphocyte-associated protein-4
CXCR4	C-X-C motif chemokine receptor 4
DCE	dynamic contrast enhanced
DEGs	differentially expressed genes
DFS	disease-free survival
DSS	disease-specific survival
DWI	diffusion weighted imaging
ECM	extracellular matrix
EF5	pentafluoroethyl-nitroimidazole
EGF	Epidermal Growth Factor
EMT	epithelial-to-mesenchymal transition
ENO1	α-Enolase
EpCAM	Epithelial Cell Adhesion Molecule
EPO	erythropoietin
FAP	fibroblast-activation protein
FFPE	formalin fixed paraffin embedded
FGF	Fibroblast Growth Factor
FSP-1	fibroblast-specific protein-1
GLUT1	Glucose transporter 1
GO	Gene ontology
GSA	Genome Sequence Archive
GSVA	Gene Set Variation Analysis
HAPs	hypoxia-activated prodrugs
HBOT	hyperbaric oxygen therapy
HCC	hepatocellular carcinoma
HIFs	hypoxia-inducible factors
iCAFs	inflammatory cancer-associated fibroblasts
ICIs	immune checkpoint inhibitors
IFN-γ	interferon-γ
IHC	immunohistochemistry
IL10	interleukin 10
IL6	interleukin 6
ILC2s	Innate lymphoid cells group 2
ITPP	Myo-inositol trispyrophosphate
KEGG	Kyoto Encyclopedia of Genes and Genomes
KM	Kaplan–Meier
L-2HG	L-isoform 2-hydroxygluterate
LASSO	least absolute shrinkage and selection operator;
LDHA	lactate dehydrogenase A
MCP-counter	Microenvironment Cell Populations-counter
MDSCs	myeloid-derived suppressor cells
MHC-I	major histocompatibility complex class I
mMICA	membrane major histocompatibility complex class 1-rleated molecule A
MMPs	matrix metalloproteinases
MRI	magnetic resonance imaging
MSI	microsatellite instability
myCAFs	myofibroblastic cancer-associated fibroblasts
NA	not applicable
NF-κB	nuclear factor kappa-light-chain-enhancer of activated B cells
NK	natural killer
NKG2D	natural killer group 2, member D
NOS2	nitric oxide synthase
NRF2	nuclear factor erythroid-related factor 2
OCT4	Octamer-binding transcription factor 4
OS	overall survival
PD-1	programmed cell death protein-1
PDAC	pancreatic ductal adenocarcinoma
pDCs	plasmacytoid dendritic cells
PDGF	Platelet-Derived Growth Factor
PD-L1	programmed cell death protein-ligand 1
PDPN	podoplanin
PET	positron emission tomography
PFS	progression-free survival
PH	proportional hazard
PHD	prolyl hydroxylase domain
POSTN	periostin
POU5F1	POU class 5 homeobox 1
PROM1	Promonin 1
PROTACs	proteolysis-targeting chimeras
PSCs	pancreatic stellate cells
PSR	Picrosirius Red
Ref	reference
ROS	reactive oxygen species
Sig.	signature
sMICA	soluble major histocompatibility complex class 1-rleated molecule A
SODs	superoxide dismutases
SOX2	SRY-box transcription factor 2)
ssGSEA	single-sample Gene Set Enrichment Analysis
STAT3	Signal Transducer and Activator of Transcription 3
Tfh	T-follicular helper cells
TGF-β	Transforming Growth Factor-β
Th	T helper
TME	tumor microenvironment
TNC	tenascin-C
TNF	Tumor Necrosis Factor
TNF-α	tumor necrosis factor-α
Tregs	regulatory T cells
TWIST1	twist family bHLH transcription factor 1
VEGF	Vascular Endothelial Growth Factor
VHL	von Hippel–Lindau
VISTA	V-domain Ig suppressor of T cell activation
vs.	versus
VWF	von Willebrand factor
